# Imaging Cellular Proliferation in Prostate Cancer with Positron Emission Tomography

**Published:** 2015

**Authors:** Hossein Jadvar

**Affiliations:** Department of Radiology, Keck School of Medicine, University of Southern California, Los Angeles, California, USA

**Keywords:** Cellular proliferation, Positron Emission Tomography, Prostate cancer

## Abstract

Prostate cancer remains a major public health problem worldwide. Imaging plays an important role in the assessment of disease at all its clinical phases, including staging, restaging after definitive therapy, evaluation of therapy response, and prognostication. Positron emission tomography with a number of biologically targeted radiotracers has been demonstrated to have potential diagnostic and prognostic utility in the various clinical phases of this prevalent disease. Given the remarkable biological heterogeneity of prostate cancer, one major unmet clinical need that remains is the non-invasive imaging-based characterization of prostate tumors. Accurate tumor characterization allows for image-targeted biopsy and focal therapy as well as facilitates objective assessment of therapy effect. PET in conjunction with radiotracers that track the thymidine salvage pathway of DNA synthesis may be helpful to fulfill this necessity. We review briefly the preclinical and pilot clinical experience with the two major cellular proliferation radiotracers, [^18^F]-3’-deoxy-3’-fluorothymidine and [^18^F]-2’-fluoro-5-methyl-1-beta-D-arabinofuranosyluracil in prostate cancer.

## Introduction

An important unmet clinical need in the imaging evaluation of prostate cancer is image-based characterization of tumor, which can facilitate clinical decision-making and patient management. Prostate cancer has a wide spectrum of biological behavior that ranges from indolent to aggressive. While indolent tumors may be managed with active surveillance, aggressive tumors will need early definitive treatment for improved patient outcome.

Positron emission tomography (PET) with various radiotracers that track particular biological pathways has been explored for the imaging evaluation of prostate cancer. These radiotracers include, but are not limited to, ^18^F-fluorodeoxygluse (glucose metabolism), ^11^C-acetate and ^11^C/^18^F-choline (cellular membrane lipogenesis), 16**a**-^18^F-fluoro-5**a**-dihydrotestosterone (androgen receptor targeting), anti-1-amino-3-^18^F-fluorocyclobutane-1-carboxylic acid (a synthetic amino acid analog), and radiotracers targeted to the gastrin-releasing peptide receptor, prostate-specific membrane antigen, and prostate stem cell antigen (1, 2). Many of the diagnostic radiotracers may also have therapeutic counterparts (theranostic pairs).

**Figure 1:**
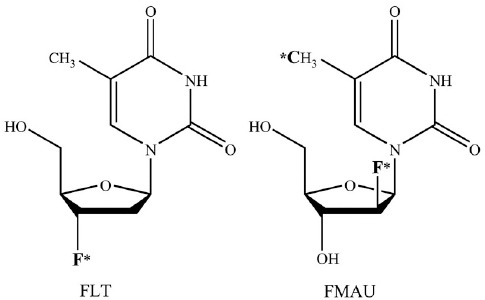
Chemical structures of ^18^F-FLT and ^18^F-FMAU (adapted from Ref. 6 and used with permission). *F denotes the position of ^18^F

Imaging cellular proliferation may provide valuable diagnostic information about the rate of tumor growth and an opportunity for objective assessment of response to treatment (3-5). PET in conjunction with radiotracers that track the thymidine salvage pathway of DNA synthesis has been studied relatively extensively for noninvasive imaging-based assessment of cellular proliferation in cancer (6, 7). Although ^11^C-thymidine was an early contender, but major limitations were encountered primarily in relation to rapid catabolism of thymidine (8-11). Further research resulted in the development of analogs that were resistant to catabolism and can be labeled with the longer half-life ^18^F (110 min) which in turn facilitates regional distribution of the tracer without the need for an on-site cyclotron. Here we briefly highlight the experience with two of these radiotracers that have been employed in preclinical and pilot clinical studies in prostate cancer, [^18^F]-3’-deoxy-3’-fluorothymidine and [^18^F]-2’-fluoro-5-methyl-1-beta-D-arabinofuranosyluracil (Figure 1).

### 

#### [^18^F]-3’-deoxy-3’-fluorothymidine

**Figure 2:**
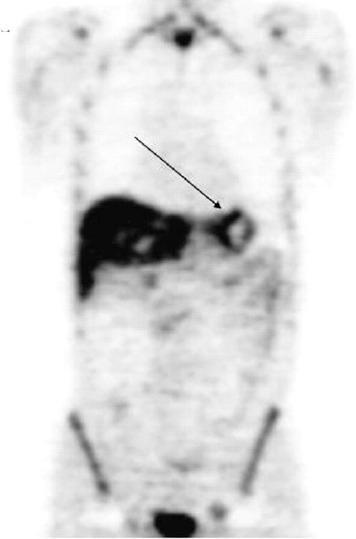
^18^F-FLT distribution in a patient with gastric cancer (arrow); high physiologic tracer localization is noted in the liver and the bone marrow with excreted urine activity in the urinary bladder (Reprinted with permission from Herman K et al. J Nucl Med 2007; 48:1945-50)

The most studied cellular proliferation PET tracer is 3’-deoxy-3’-fluorothymidine(^18^F-FLT) which is phosphorylated by thymidine kinase 1 (TK1), retained in proliferating cells without DNA incorporation, and can be described by a three-compartment model (12-15). Normal biodistribution of ^18^F-FLT demonstrates relatively high uptake in the liver and the bone marrow with urinary bladder receiving the highest dose through renal excretion (16) (Figure 2). Kukuk et al. from Germany investigated the pharmacokinetics of ^18^F-FLT, ^18^F-fluorodeoxyglucose (^18^F-FDG), and ^11^C-choline in 2 hormone independent (PC-3, DU145) and 2 hormone-dependent (CWR22, PAC120) prostate cancer xenograft mouse models using PET (17). Both ^18^F-FLT and ^18^F-FDG showed the highest uptake in PC-3 tumors. However, while ^18^F-FDG uptake in CWR22 tumor was high and decreased markedly after androgen deprivation therapy, the uptake of^18^F-FLT was insufficient to provide reliable information on response to therapy. Conversely, an earlier study reported that ^18^F-FLT uptake in the implanted CWR22 tumor was markedly reduced after castration or diethylstilbestrol treatment (18).

In another preclinical micro PET study, a significant decline in ^18^F-FLT uptake was noted in the 22Rv1 hormone-refractory prostate tumors implanted in athymic mice after treatment with docetaxel (19). Interestingly, in this study, changes in prostate-specific antigen concentration in the cell medium and ^18^F-FDG uptake in response to treatment were minimal. The authors concluded that ^18^F-FLT is a promising tracer for early assessment of anticancer therapy with docetaxel in patient with hormone refractory prostate cancer. Therefore, while it appears that ^18^F-FLT may be helpful in the evaluation of treatment response in prostate cancer, the exact utility of ^18^F-FLT in this context remains unsettled, especially given the fact that there is high physiologic localization of the radiotracer in the normal bone marrow that is the most common site for prostate tumor metastases.

#### [^18^F]-2’-fluoro-5-methyl-1-beta-D-arabinofuranosyluracil

This thymidine analog is phosphorylated by thymidine kinase and incorporated in the DNA. The unlabeled compound (abbreviated as FMAU) was originally of clinical interest as an anticancer and an antiviral drug when used in pharmacological dose (20). Tehrani et al. showed that this thymidine analog is preferentially phosphorylated by the mitochondrial thymidine kinase 2 (TK2) in comparison to the cytosolic TK1 (21). In tracer doses, this agent can be labeled with ^11^C or ^18^F and as such are useful for imaging DNA synthesis and tumor proliferation (22-26). It has also been used for imaging reporter gene expression using the herpes simplex virus type 1 thymidine kinase (HSV-tk1) system (27-30). Recently, an automated cGMP-compliant radiosynthesis of FMAU has been described (31).

Pharmacokinetic studies have shown that ^14^C-FMAU behaves very similar to the pyrimidine nucleoside, thymidine, with respect to cellular uptake velocity, saturability of cellular incorporation, and intracellular metabolite pools and is reflective of tumor cell division (32). A recent report from our laboratory at the University of Southern California showed that ^11^C-FMAU uptake in a dog brain tumor model correlated with tumor growth rate and could be well described by a three-compartment kinetic model (33). The adequacy of three-compartment model has also been shown for ^18^F-FMAU (34). One study comparing the L-isomer with the D-isomer showed higher accumulation of the D-isomer in both fast growing H441 (byafactorofabout7.74), and slow growing H3255 (by a factor of about 3.37) human lung cancer cell lines (35). Of note, these values were significantly higher than those for the L-isomer ^18^F-FMAU and ^18^F-FLT.

**Figure 3:**
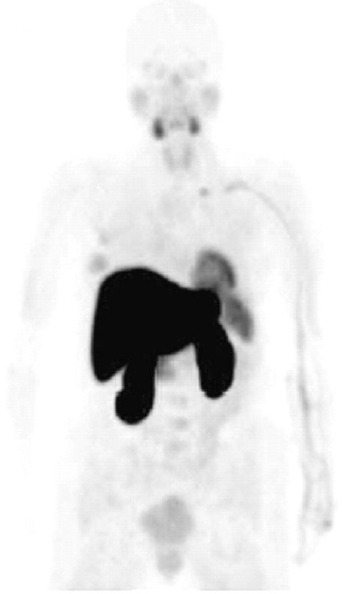
Normal biodistribution of ^18^F-FMAU in human; note the relatively high tracer uptake in the liver and the renal cortex, moderate uptake in the salivary glands, heart, and spleen and relatively low uptake in the bone marrow (adapted from Ref. 6 and used with permission)

Initial imaging-based biodistribution of ^18^F-FMAU in normal dogs have shown that ^18^F-FMAU is resistant to degradation, and is selectively retained in DNA (36). ^18^F-FMAU shows little accumulation in bone (a common site for metastasis from prostate cancer) that renders it a potentially ideal PET radiotracer for imaging DNA synthesis in prostate cancer (37) (Figure 3). Recently our laboratory showed that there may be an association between androgen signaling and thymidine metabolism and that ^18^F-FMAU PET may be useful in prostate tumor characterization (38). One possibility may be the androgen control of mitochondrial function that may include TK2 enzymatic activity (39).

A pilot observational study of ^18^F-FMAU PET in three men with prostate cancer confirmed tumor retention of ^18^F-FMAU in local prostate recurrence, and in metastatic lesions with barely visible activity in the urinary bladder and the normal bone (in prostate recurrence: tumor-to-background pelvis activity ratio of 2.3-6.3; in bone metastasis: tumor-to-background normal bone activity ratio of 2.4-3.1) (40). Moreover, on average, 95% of the blood activity cleared within 10 minutes post ^18^F-FMAU administration, and about 70% of the activity in the urine was intact ^18^F-FMAU at 60 minutes post injection. We have also recently initiated a pilot study to assess the potential utility of ^18^F-FMAU in image-targeted biopsy using sophisticated software-based fusion of PET, transrectal ultrasound and magnetic resonance imaging of the prostate gland. Such hybrid imaging methodology may allow for improved localization and characterization of tumors for targeted biopsy and focal therapy. Additional applications may include the use of ^18^F-FMAU in the assessment of treatment response, and prognosis in men with metastatic castrate-resistant prostate cancer.

Although other substituted 2’-[^18^F]fluro-2’-deoxy-arabinofuranosyluracil derivatives such as 2’-deoxy-2’-[^18^F]fluoro-5-bromo-1-beta-D-arabinofuranosyluracil (^18^F-FBAU), 2’- deoxy-2’[^18^F]fluro-5-chloro-1-beta-D-arabinofuranosyl-uracil (^18^F-FCAU), 2’-deoxy-2’-^18^F-fluoro-5-fluoro-1-beta-D-arabinofuranosyluracil (^18^F-FFAU), and others have also been synthesized, but their exact clinical utility and potential competitive advantage over ^18^F-FLT and ^18^F-FMAU will need further exploration (41, 42).

## Conclusions

Imaging cellular proliferation in prostate cancer allows for personalized precision care in men with prostate cancer. Imaging-based tumor characterization allows for improved clinical decision-making through management stratification that may lead to enhanced patient outcome, decreased adverse events and lower cost of care. Additional prospective investigations of imaging cellular proliferation in prostate cancer are warranted.

## References

[1] Jadvar H (2012). Molecular imaging of prostate cancer: PET radiotracers. AJR Am J Roentgenol.

[2] Yu CY, Desai B, Ji L, Groshen S, Jadvar H (2014). Comparative performance of PET tracers in biochemical recurrence of prostate cancer: a critical analysis of literature. Am J Nucl Med Mol Imaging.

[3] Mankoff DA, Shields AF, Krohn KA (2005). PET imaging of cellular proliferation. Radiol Clin North Am.

[4] Couturier O, Leost F, Campone M, Cartlier T, Chatal JF, Hustinx R (2005). Is 3’-deoxy-3’-[18F]fluorothymidine ([18F]-FLT) the next tracer for routine clinical PET after [18F]-FDG?. Bull Cancer.

[5] Nimmagadda S, Shields AF (2008). The role of DNA synthesis imaging in cancer in the era of targeted therapeutics. Cancer Metastasis Rev.

[6] Bading JR, Shields AF (2008). Imaging of cell proliferation: status and prospects. J Nucl Med.

[7] Tehrani OS, Shields AF (2013). PET imaging of proliferation with pyrimdines. J Nucl Med.

[8] Shields AF, Mankoff D, Graham MM, Zheng M, Kozawa SM, Link JM (1996). Analysis of 2-carbon-11-thymidine blood metabolites in PET imaging. J Nucl Med.

[9] Shields AF, Mankoff DA, Link JM, Graham MM, Eary JF, Kozawa SM (1998). Carbon-11-thymidine and FDG to measure therapy response. J Nucl Med.

[10] Mankoff D, Shields AF, Link JM, Graham MM, Muzi M, Pterson LM (1999). Kinetic analysis of 2-[11C]thymidine PET imaging studies: validation studies. J Nucl Med.

[11] Mankoff DA, Shield AF, Graham MM, Link JM, Eary JF, Krohn KA (1998). Kinetic analysis of 2-[carbon- 11]thymidine PET imaging studies: compartmental model and mathematical analysis. J Nucl Med.

[12] Shields AF, Grierson JR, Muzik O, Stayanoff JC, Lawhorn-Crews JM, Obradovich JE (2002). Kinetics of 3’-deoxy-3’-[F- 18]fluorothymidine uptake and retention in dogs. Mol Imaging Biol.

[13] Shields AF, Briston DA, Chandupatla S, Douglas KA, Lawhorn-Crews J, Collins JM (2005). A simplified analysis of [18F]3’- deoxy-3’-fluorthymidine metabolism and retention. Eur J Nucl Med Mol Imaging.

[14] Shields AF, Grierson JR, Dohmen BM, Machulla HJ, Stananoff JC, Lawhorn-Crews JM (1998). Imaging proliferation in vivo with [F- 18]FLT and positron emission tomography. Nat Med.

[15] Grierson JR, Shields AF (2000). Radiosynthesis of 3′-deoxy-3′-[18F]fluorothymidine:[18F]FLT for imaging of cellular proliferation in vivo. Nucl Med Biol.

[16] Vesselle H, Grierson J, Peterson LM, Muzi M, Mankoff DA, Krohn KA (2003). ^18^F-fluorothymidine radiationdosimetry in human PET imaging studies. J Nucl Med.

[17] Kukuk D, Reischl G, Raguin O, Wiehr S, Judenhofer MS, Calaminus C (2011). Assessment of PET tracer uptake in hormone-independent and hormone-dependent xenograft prostate cancer mouse models. J Nucl Med.

[18] Oyama N, Ponde D, Dence C, Kim J, Tai YC, Welch MJ (2004). Monitoring of therapy in androgen dependent prostate tumor model by measuring tumor proliferation. J Nucl Med.

[19] Oyama N, Hasegawa Y, Kiyono Y, Kobayashi M, Fujibayashi Y, Ponde DE (2011). Early response assessment in prostate carcinoma by 18F-fluorothymidine following anticancer therapy with docetaxel using preclinical tumor models. Eur J Nucl Med Mol Imaging.

[20] Fanucchi MP, Leyland-Jones B, Young CW, Burchenal JH, Watanabe KA, Fox JJ (1985). Phase I trial of 1-(2’-deoxy- 2’-fluoro-1-beta-D-arabinofuranosyl)-5-methyluracil (FMAU). Cancer Treat Rep.

[21] Tehrani OS, Douglas KA, Lawhorn-Crews JM, Shields AF (2008). Tracking cellular stress with labeled FMAU reflects changes in mitochondrial TK2. Eur J Nucl Med Mol Imaging.

[22] Conti PS, Alauddin MM, Fissekis JR, Schmall B, Watanabe KA (1995). Synthesis of 2’-fluoro-5-[11C]-methyl-1-beta-D-arabinofuranosyluracil ([11C]-FMAU): a potential nucleoside analog for in vivo study of cellular proliferation with PET. Nucl Med Biol.

[23] Bading JR, Shahinian AH, Bathija P, Conti PS (2000). Pharmacokinetics of the thymidine analog 2’-fluoro-5-[(14)C]-methyl-1-beta-D-arabinofuranosyluracil ([(14)C]FMAU) in rat prostate tumor cells. Nucl Med Biol.

[24] Wang H, Oliver P, Nan L, Wang S, Wang Z, Rhie JK (2002). Radiolabeled 2’-fluorodeoxyuracil-beta-D-arabinofuranoside (FAU) and 2’-fluoro-5-methyldeoxyuracil-beta -D-arabinofuranoside (FMAU) as tumor-imaging agents in mice. Cancer Chemo ther Pharmacol.

[25] Lu L, Samuelsson L, Bergstrom M, Sato K, Fasth KJ, Langstrom B (2002). Rat studies comparing 11C-FMAU, 18F-FLT, and 76Br-BFU as proliferation markers. J Nucl Med.

[26] Mangner TJ, Klecker RW, Anderson L, Shields AF (2003). Synthesis of 2’-deoxy-2’-[18F]fluoro-beta-D-arabinofuranosyl nucleosides, [18F]FAU, [18F]FMAU, [18F]FBAU and [18F]FIAU, as potential PET agents for imaging cellular proliferation. Synthesis of [18F]labelled FAU, FMAU, FBAU, FIAU. Nucl Med Biol.

[27] de Vries EF, van Waarde A, Harmsen MC, Mulder NH, Vaaburg W, Hospers GA (2000). [(11)C]FMAU and [(18)F]FHPG as PET tracers for herpes simplex virus thymidine kinase enzyme activity and human cytomegalovirus infections. Nucl Med Biol.

[28] Alauddin MM, Shahinian A, Gordon EM, Conti PS (2002). Evaluation of 2’-deoxy-2’- fluoro-5-methyl-1-beta-D-arabinofurasyluracil as a potential gene imaging agent for HSV-tk expression in vivo. Mol Imaging.

[29] Alauddin MM, Shahinian A, Park R, Tohme M, Fissekis JD, Conti PS (2004). Synthesis and evaluation of 2’-deoxy-2’-18F-fluoro-5-fluoro-1-beta-D-arabinofuranosyluracil as a potential PET imaging agent for suicide gene expression. J Nucl Med.

[30] Kang KW, Min JJ, Chen X, Gambhir SS (2005). Comparison of [14C]FMAU, [3H]FEAU, [14C]FIAU, and [3H]PCV for monitoring reporter gene expression of wild type and mutant herpes simplex virus type I thymidine kinase in cell culture. Mol Imaging Biol.

[31] Li Z, Cai H, Conti PS (2011). Automated synthesis of 2’-deoxy-2’-[18F]fluoro-5-methyl- 1-b-D-arabinofuranosyluracil ([18F]-FMAU) using a one reactor radiosynthesis module. Nucl Med Biol.

[32] Bading JR, Shahinian AH, Vail A, Bathija P, Koszalka GW, Koda RT (2004). Pharmacokinetics of the thymidine analog 2’-fluoro-5-methyl-1-beta-D-arabinofuranosyluracil (FMAU) in tumor bearing rats. Nucl Med Biol.

[33] Conti PS, Bading JR, Mouton PP, Links JM, Alauddin MM, Fissekis JD (2008). In vivo measurement of cell proliferation in canine brain tumor using C-11-labeled FMAU and PET. Nucl Med Biol.

[34] Tehrani OS, Muzik O, Heilbrun LK, Douglas KA, Lawhorn-Crews JM, Sun H (2007). Tumor imaging using 1-(2’-deoxy-18Ffluoro-beta-D-arabinofuranosyl) thymine and PET. J Nucl Med.

[35] Nishii R, Volgia AY, Mawlawi O, Mukhopadhyay U, Pal A, Bommann W (2008). Evaluation of 2’-deoxy-2’-[(18)F]fluoro-5- methyl-1-beta-L:-arabinofuranosyluracil ([(18)F]-L:-FMAU) as a PET imaging agent for cellular proliferation: comparison with [(18)F]-D:-FMAU and [(18)F]FLT. Eur J Nucl Med Mol Imaging.

[36] Sun H, Mangner TJ, Collins JM, Muzik O, Douglas K, Shields AF (2005). Imaging DNA synthesis in vivo with 18F-FMAU and PET. J Nucl Med.

[37] Shields AF (2006). Positron emission tomography measurement of tumor metabolism and growth: its expanding role in oncology. Mol Imaging Biol.

[38] Jadvar H, Yap LP, Park R, Li Z, Chen K, Hughes L (2012). [18F]-2’-fluoro-5-methyl-1-beta-Darabinofuranosyluracil (18F-FMAU) in prostate cancer: initial preclinical observations. Mol Imaging.

[39] Doeg KA, Polomski LL, Doeg LH (1972). Androgen control of mitochondrial and nuclear DNA synthesis in male sex accessory tissue of castrate rats. Endocrinology.

[40] Sun H, Sloan A, Mangner TJ, Vaishamayan U, Muzik O, Collins JM (2005). Imaging DNA synthesis with [18F]FMAU and positron emission tomography in patients with cancer. Eur J Nucl Med Mol Imaging.

[41] Alauddin MM, Shahinian A, Gordon EM, Conti PS (2004). Direct comparison of radiolabeled probes FMAU, FHBG, and FHPG as PET imaging agents for HSV1-tk expression in a human breast cancer model. Mol Imaging.

[42] Cai H, Li Z, Conti PS (2011). The improved syntheses of 5-substituited 2’-[18F]fluoro-2’-deoxy-arabinofuranosyluracil derivatives ([18F] FAU, [18F] FEAU, [18F] FFAU, [18F] FCAU, [18F] FBAU and [18F] FIAU) using a multistep one-pot strategy. Nucl Med Biol.

